# A pilot study on the prevalence of DNA palindromes in breast cancer genomes

**DOI:** 10.1186/s12920-016-0232-3

**Published:** 2016-12-05

**Authors:** Sandeep Subramanian, Srilakshmi Chaparala, Viji Avali, Madhavi K. Ganapathiraju

**Affiliations:** 1 Department of Biomedical Informatics, University of Pittsburgh, 5607 Baum Blvd, Suite 522, Pittsburgh, PA 15206 USA; 2 Language Technologies Institute, Carnegie Mellon University, Pittsburgh, PA 15213 USA

## Abstract

**Background:**

DNA palindromes are a unique pattern of repeat sequences that are present in the human genome. It consists of a sequence of nucleotides in which the second half is the complement of the first half but appearing in reverse order. These palindromic sequences may have a significant role in DNA replication, transcription and gene regulation processes. They occur frequently in human cancers by clustering at specific locations of the genome that undergo gene amplification and tumorigenesis. Moreover, some studies showed that palindromes are clustered in amplified regions of breast cancer genomes especially in chromosomes (chr) 8 and 11. With the large number of personal genomes and cancer genomes becoming available, it is now possible to study their association to diseases using computational methods. Here, we conducted a pilot study on chromosomes 8 and 11 of cancer genomes to identify computationally the differentially occurring palindromes.

**Methods:**

We processed 69 breast cancer genomes from The Cancer Genome Atlas including serum-normal and tumor genomes, and 1000 Genomes to serve as control group. The Biological Language Modelling Toolkit (BLMT) computes palindromes in whole genomes. We developed a computational pipeline integrating BLMT to compute and compare prevalence of palindromes in personal genomes.

**Results:**

We carried out a pilot study on chr 8 and chr 11 taking into account single nucleotide polymorphisms, insertions and deletions. Of all the palindromes that showed any variation in cancer genomes, 38% of what were near breast cancer genes happened to be the most differentiated palindromes in tumor (i.e. they ranked among the top 25% by our heuristic measure).

**Conclusions:**

These results will shed light on the prevalence of palindromes in oncogenes and the mutations that are present in the palindromic regions that could contribute to genomic rearrangements, and breast cancer progression.

## Background

Most eukaryotic genomes contain repeat sequences in their DNA and nearly half of the human genome is covered by various types of repeats. DNA palindromes are a unique pattern of repeat sequences that are found in both prokaryotes and eukaryotes. It consists of a sequence of nucleotides in which the second half is the complement of the first half but appearing in reverse order [1, 2]. For example, 5′-GTTAG|CTAAC-3′ is a DNA palindrome. Proteins such as restriction enzymes and transcription factors that function as dimers often recognize the two-fold symmetry of palindromic sequences and bind to them. This two-fold symmetry helps to increase the affinity and specificity of interaction between DNA and proteins [3, 4]. The ability of a palindromic sequence to fold around its midpoint to form a double strand with itself enables it to form a secondary structure called cruciform or hairpin structure. These secondary structures are known to be associated with chromosomal translocations and rearrangements that could contribute to errors in DNA replication and gene expression leading to human diseases such as male infertility and thalassemia [4–7]. Recently, researchers discovered that the palindromic GOLGA8 regions might be contributing to microdeletions in chr 15 that are associated with schizophrenia, autism, intellectual disability and epilepsy [8].

DNA Palindromes occur frequently in human cancer cell lines, including medulloblastoma, breast cancer and colorectal adenocarcinoma. A microarray based approach called Genome-wide Analysis of Palindrome Formation (GAPF) detected a non-random distribution of palindromes in human cancers including breast cancer and colon cancer genomes; palindromes tend to cluster at specific regions that undergo gene amplification [9]. Long palindromes are associated with gene amplification and genomic instability in cancers. A consistent formation was also observed at a microRNA gene called bic/miR-155 that is associated with tumor development. Further, palindromes and short tandem repeats were found in APC gene that is associated with colorectal polyps; polyps are precancerous lesions that will develop into colorectal cancer at a later stage. These studies further suggest that palindrome formation may influence the tumor formation and cancer development [9–12]. GAPF positive regions are those regions that are enriched in cancer cell lines relative to the normal human fibroblasts. When at least three such regions are present, it is called a cluster. GAPF positive regions are clustered in breast cancer genomes especially in chr 8 and chr 11. These chromosomes are also susceptible to DNA amplification and chromosomal aberrations, which are correlated to overexpression of oncogenes and to tumorigenesis in breast cancer [13–17]. Amplification events in chr 11 that are associated with oncogenes are also reported in breast, ovarian, and lung cancers [18]. These aspects support a possible role of palindromes in cancers mediated by DNA amplification [13–15].

The availability of whole genome sequences of individuals makes it possible to study computationally the prevalence of palindromes and their relative abundance in various genomic locations. By studying the differential distribution of palindromes in genomes of cancer patients and tumors, we may be able to shed light on their influence on gene amplifications and genomic rearrangements, and their relevance in cancer. To our knowledge, there have been no studies of DNA palindromes in personal genomes (i.e. a genome incorporating variants of an individual from 1000 Genomes, cancer genomes, etc.) except for one preliminary study of an early draft of the reference genome [4].

We developed a suite of tools to identify palindromes efficiently in personal genomes and to compare them across multiple genomes. In this study, we present our analysis of the palindrome distribution and changes in chr 8 and chr 11 of 69 breast cancer genomes (normal and tumor) and compare them in relation to genomes from the 1000 Genomes project [19].

## Methods

### Data

We analysed 69 matched tumor-normal breast cancer genomes from The Cancer Genome Atlas (TCGA), and the same number of personal genomes from 1000 Genomes project to serve as control. Variant files corresponding to whole genomes from TCGA are available to us through the Pittsburgh Genome Resource Repository (PGRR). PGRR provides a mechanism for University of Pittsburgh investigators to access and use TCGA datasets from a central location using common tools and platforms. We analysed 69 matched tumor and normal whole genomes of breast cancer, in this pilot study; we also restricted our focus on chr 8 and chr 11. To serve as a control group, we analysed the same number of whole genomes from the 1000 Genomes which contains genomes of 2504 individuals overall [19]. We refer to these three types of genomes as tumor, normal and 1000 g genomes.

These genomes are available as variants in comparison to reference genome build GRCh37; the personal genome sequences are constructed by incorporating the corresponding variants into the reference genome. If a variant is multi-allelic, the first allele is incorporated into the genome and the second allele is considered while post-processing.

We used the Biological Language Modelling Toolkit (BLMT) (version 2) to identify palindromes in the human genomes [20]. BLMT pre-processes the whole genome sequence into suffix arrays and then computes the longest common prefix array, which make searching for patterns like palindromes very efficient. BLMT computes palindromes that are perfectly palindromic in the central eight bases, and expands it on both arms until it remains palindromic, but allowing for a user-specified number of mismatches. We set this mismatch tolerance to be four. The extension is constrained to be of same length on either side (i.e. insertion of unmatched base on only one side is not allowed).

The position of a palindrome in a personal genome and its position in the reference genome are not the same because of deletions and insertions preceding the palindrome. To align the corresponding palindromes between reference and personal genomes, we keep track of the offsets introduced due to insertions and deletions until the location of that palindrome.

We create a master list of all palindromes in all personal genomes, indexed by their mid-point as per its location in the reference genome.

We looked for palindromes that changed significantly in tumor samples but not in normal or 1000 g samples. We did this using two different heuristics – the first sorts palindromes in decreasing order of the difference between number of changes in tumor samples and normal samples normalized by the number of changes in 69 of the 1000 g samples. With this, we identify palindromes that have varied more in tumor samples than normal ones accounting for variants in our control group. Normalizing by the number of changes in the 1000 g data penalizes palindromes that also vary in the general population. Next, we computed the *t*-test (with unequal variances) of changes in tumor vs 1000 g and normal vs 1000 g and then computed their ratio. To compute the *t*-test statistic between the palindrome length changes in tumor samples vs 1000 g, we ran 100 experiments each with 69 randomly sampled subset of 1000 g, and computed the average of the *t*-test statistic. Using the *t*-test statistic makes our metric sensitive to the extent of change in samples, which is evident from its formulation below.

1 $$ t=\frac{\overline{X_1}-\overline{X_2}}{s_{\overline{X_1}-\overline{X_2}}} $$

2 $$ {s}_{\overline{X_1}-\overline{X_2}} = \sqrt{\frac{{s_1}^2}{n_1}+\frac{{s_2}^2}{n_2}} $$

where $\overline{x_l}$, *s*_*i*_^2^ and *n*_*i*_ are the mean, the unbiased estimator of the variance and the number of participants in the two samples.

We used annovar to annotate palindrome locations with gene regions [17]. We compiled the list of oncogenes and breast cancer related genes from Cancer Genetics Web (http://www.cancer-genetics.org/).

## Results

**Fig. 1:**
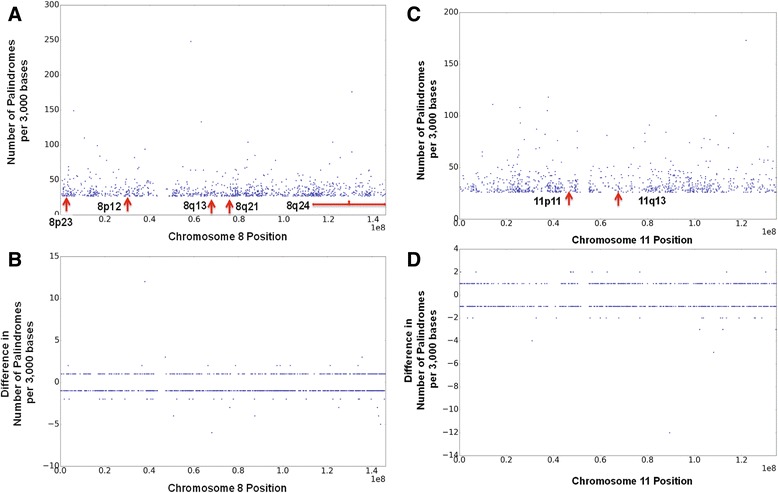
Regions that have highest density of palindromes. The number of the palindromes per 3000 bases is computed. The top 2% of the windows that are most dense with palindromes are shown for chromsomes 8 and 11 in (**a**) and (**c**). Corresponding numbers were computed for one of the TCGA samples, and the difference with respect to reference genome (tumor – reference) are shown in (**b**) and (**d**)

We computed the palindromes in the human reference genome using Biological Language Modeling Toolkit. We focused our analysis on chr 8 and chr 11 in this pilot study. In the reference genome, we found that overall there are a total of 685,064 palindromes in chr 8 and 600,274 in chr 11. On an average, there are 12 palindromes per 3000 bases, but some regions have more than 100 palindromes per 3000 bases. In the 2504 genomes of 1000 g, there are 684,211 palindromes in chr 8 on an average, and 599,308 in chr 11. Of these, about 28,000 palindromes of chr 8 had variants in them, some of which altered their length. In chr 11 about 25,000 had variants. Density of palindromes was comparable in cancer genomes. Figure 1 shows the density of palindromes per 3000 bases and the difference in density between the reference genome and one random TCGA sample, for chromosome 8 (Fig. 1a and b) and 11 (Fig. 1c and d).

**Fig. 2:**
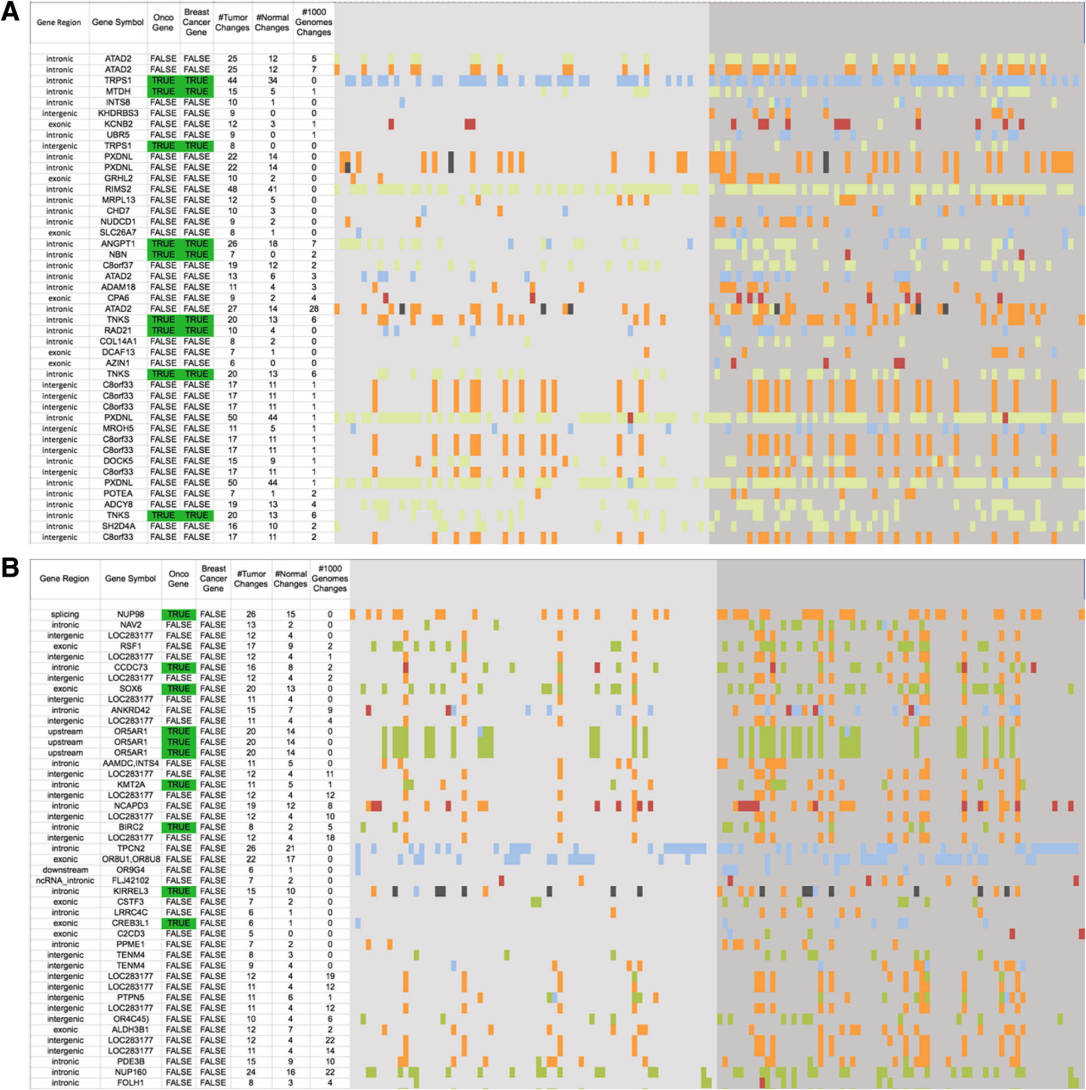
List of Palindromes. List of palindromes in (**a**) chr8 and (**b**) chr11 with significant changes in tumor and normal samples in TCGA brca dataset and minimum changes in 1000 Genomes with the following color coding: absence of the palindrome (*orange*), bigger than in the reference genome (*pale blue*), smaller than in the reference genome (*pale green*), perfect palindrome in the reference genome which is now a near palindrome with a single mismatch the central eight bases (*dark grey*) and palindrome that had the same length as in the reference genome but had a variant (*burgundy*). The left side of the figure represents normal samples and the right side tumor samples. Oncogenes and/or brca genes are highlighted in *green*

**Fig. 3:**
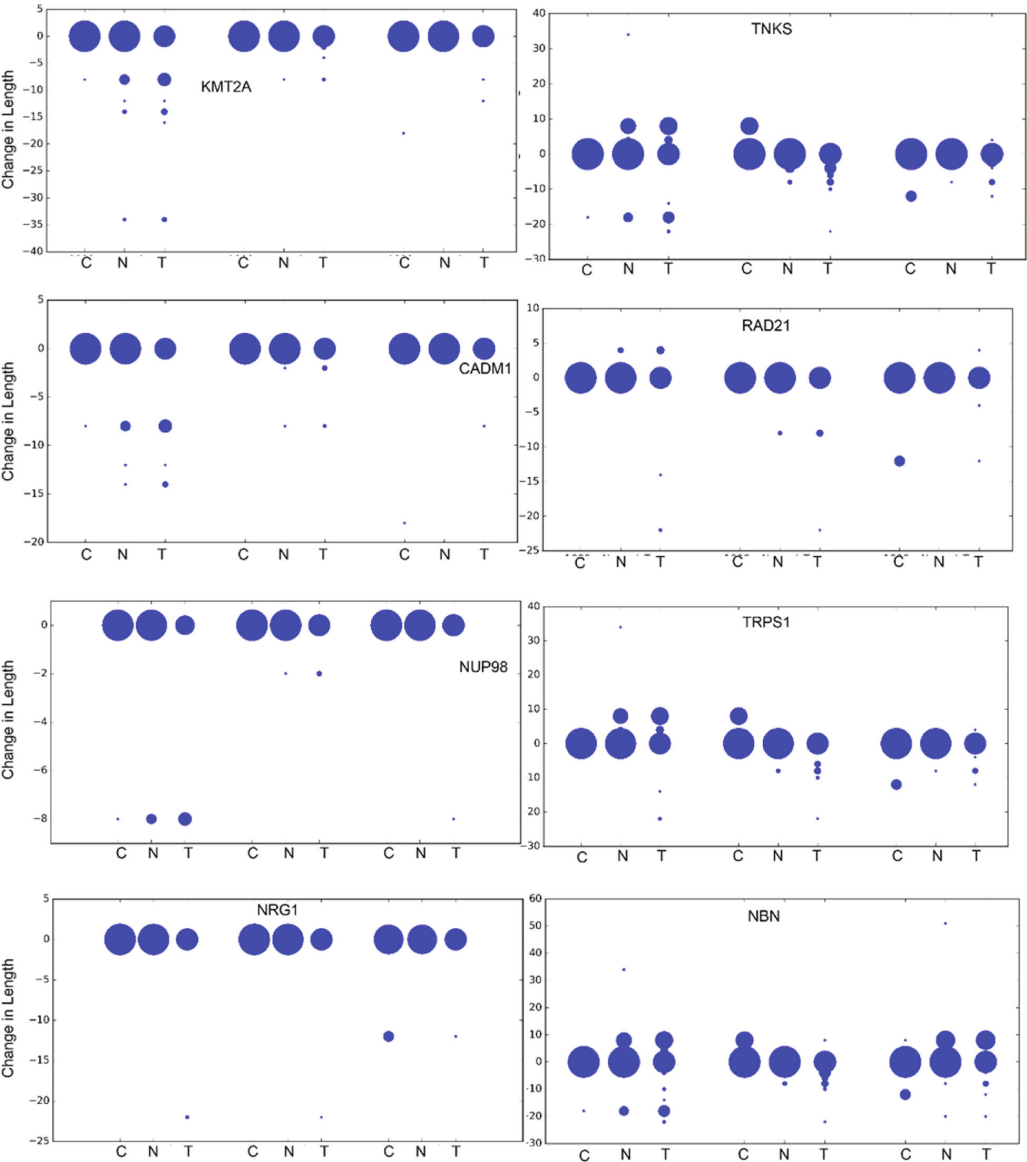
Changes in specific palindromes in eight genes. Three palindromes that have changed most significantly are shown for eight genes (KMT2A, TNKS, CADM1, RAD21, NUP98, TRPS1, NRG1 and NBN). For each palindrome of any gene, its difference in the length in comparison to reference genome are shown separately for 1000 g, normal and tumor genomes. The size of the circle is proportional to the number of samples out of the 69 considered in each set. C represents Control, N represents Normal and T represents Tumor in the figure

We created a master list of all palindromes that occur in any of the personal genomes indexed by their corresponding location in the reference genome. For each of these palindromes, we analyzed whether there is a difference in its presence in tumor vs normal or 1000 g. Figure 2a and b present palindromes that rank the highest according to our first heuristic (seeMethods). It also highlights changes that occurred within these palindromes across the TCGA samples in color-coded manner. Figure 3 present eight genes of chr 8 and chr 11 that contain specific palindromes that are significantly altered in tumors. Table 1A and B contains palindromes that rank the highest according to our second heuristic that computes the ratio of the *t*-test statistics of tumor vs normal.

**Table 1:** Table of statistical significance values for Chr8 (a) and Chr11 (b). Genes with their regions, whether the gene is oncogene or brca gene with the statistical significance are shown in separate columns

Gene region	Gene symbol	Oncogene	Breast cancer gene	Odds ratio
Chromosome 8				
intronic	SPIDR	FALSE	FALSE	12.5
intronic	EMC2	FALSE	FALSE	4.6
intergenic	CASC9	FALSE	FALSE	3.9
intronic	RAD54B	FALSE	FALSE	3.6
intronic	SH2D4A	FALSE	FALSE	3.6
intronic	MAK16	FALSE	FALSE	3.5
intronic	UBXN2B	FALSE	FALSE	3.4
intronic	KIAA1429	FALSE	FALSE	3.3
intronic	UBXN2B	FALSE	FALSE	3.0
exonic	SLC26A7	FALSE	FALSE	3.0
intronic	KIAA0196	FALSE	FALSE	2.8
intronic	POTEA	FALSE	FALSE	2.8
intergenic	UNC5D	FALSE	FALSE	2.7
intronic	CA1	FALSE	FALSE	2.7
intronic	EIF3E	TRUE	TRUE	2.5
intronic	CPQ	FALSE	FALSE	2.5
exonic	USP17L7	FALSE	FALSE	2.5
intronic	SH2D4A	FALSE	FALSE	2.5
exonic	C8orf37	FALSE	FALSE	2.5
intronic	SDC2	FALSE	FALSE	2.4
exonic	DCAF13	FALSE	FALSE	2.4
intronic	CPQ	FALSE	FALSE	2.4
intronic	CCAR2	TRUE	FALSE	2.4
ncRNA_intronic	LOC392232	FALSE	FALSE	2.4
exonic	GRHL2	FALSE	FALSE	2.4
intronic	TRPS1	TRUE	TRUE	2.3
Chromosome 11				
intergenic	LOC283177	FALSE	FALSE	17.0
intronic	NFRKB	FALSE	FALSE	14.9
intronic	DCDC5	FALSE	FALSE	6.0
intronic	C2CD3	FALSE	FALSE	5.0
intergenic	LOC283177	FALSE	FALSE	4.2
intergenic	LOC102724301	FALSE	FALSE	4.1
intergenic	LOC283177	FALSE	FALSE	3.9
intergenic	FAM86C2P	FALSE	FALSE	3.9
intronic	KIRREL3	TRUE	FALSE	3.8
intronic	NUCB2	FALSE	FALSE	3.5
intronic	MYO7A	FALSE	FALSE	3.4
intergenic	MIR8068	FALSE	FALSE	3.2
intergenic	OR4A5	FALSE	FALSE	3.1
intronic	CADM1	TRUE	FALSE	3.0
intergenic	LOC283177	FALSE	FALSE	3.0
exonic	NXPE1	FALSE	FALSE	2.9
intronic	CWF19L2	FALSE	FALSE	2.8
intronic	PRCP	FALSE	FALSE	2.8
intergenic	LOC283177	FALSE	FALSE	2.7
intergenic	LOC283177	FALSE	FALSE	2.7
intronic	MYO7A	FALSE	FALSE	2.6
downstream	OR51B4	FALSE	FALSE	2.6
intergenic	LOC283177	FALSE	FALSE	2.5

## Discussion

DNA Palindromes were shown to be distributed non-randomly in breast cancer cell lines [11]. In addition, they were also found to be clustered in gene amplicons chr 8 and chr 11, specifically, 8p12, 8q21, 8p23, 11q12 and 11q13 in breast cancer when associated with copy-number gains and amplifications [10, 21]. We analysed palindromes that have changed significantly in breast cancer genomes (tumor and/or normal) in comparison to palindromes in 1000 genomes. Of all the palindromes that showed any variation in cancer genomes, 38% of what were near breast cancer genes happened to be the most differentiated palindromes in tumor (i.e. they ranked among the top 25% by first heuristic measure).

An intronic palindrome that is associated with one of the breast cancer gene (brca) NBN, that is in 8q21 region, shows significant changes in tumors ie., changes in seven tumors but no change in any normal samples. NBN mutations have shown to be associated with chromosomal rearrangements and instability, with increased risk for cancers including breast cancer. It encodes nibrin that is involved in DNA damage and repair [22]. One of the palindromes in intronic region of RAD21 has a germline variant; RAD21 plays a role in double strand break repair mechanism and is associated with multiple cancers [23]. Tumors have a tendency to accumulate mutations that could disrupt DNA repair, which leads to DNA damage. Therefore, palindrome associations in NBN and RAD21 may be important to understand the role of these proteins in DNA damage and repair [24]. Another gene TRPS1 shows that there are 3 intergenic palindromes that changed significantly in tumors. One of these intergenic palindromes is absent in many tumor samples whereas there are no changes observed in normal samples; other intergenic palindromes got smaller in tumors with no changes in normal samples. TRPS1 gene belongs to the family of transcription factors and may have role in regulating cell proliferation and growth [25]. This gene is localized in chr8q23-24.1, and this region is known to be highly amplified in breast and prostate cancers. Palindromes in SPIDR are significantly altered in tumors (Table 1A). SPIDR is a scaffolding protein that is involved in homologous recombination repair mechanism and is shown to have breast and ovarian cancer susceptibility [26].

Through similar analysis of palindromes in chr 11, we found that palindromes in oncogenes NUP98 and KMT2A have significant changes in tumors when compared to normal and 1000 genomes. All palindromes observed in NUP98 were in the intronic region, and three intronic, three exonic and one intergenic palindromes were observed in KMT2A gene. In NUP98, one palindrome is completely absent in tumors, whereas an intronic palindrome in KMT2A got bigger. NUP98 is a nuclear pore gene that is required for induction of p53 target genes and is associated with cancers such as hepatocellular carcinoma. KMT2 genes are most frequently mutated genes in various cancers. KMT2A is present in chr11q23 region that undergo frequent genomic rearrangements, and somatic mutations in KMT2A are associated with leukaemia [27]. We also found that CADM1 has an intronic palindrome that shows significant changes in tumors and loss of CADM1 expression is associated with poor prognosis in breast cancer patients and identified as metastasis susceptibility gene in breast cancer [28].

## Conclusion

Many palindromes are significantly different in tumors when compared to serum-normal and 1000 Genomes data. These findings will further support the role of palindromes in cancers including breast cancer. We believe that further experimental analysis of these palindrome variations will help to identify the effect of these variants on genomic rearrangements and downstream effects such as gene expression. New palindromes that are formed because of variants may even serve as binding sites to transcription factors [29], leading to abnormal gene expression. These findings will help to identify the palindromes that could be potential biomarkers for breast cancer in the future. We limited our variant analysis to SNPs, insertions and deletions in this study but we are planning to include copy number variations in future work. This is a pilot study to highlight a very important question in cancer genomics that is amenable to study by computational methods by leveraging large amounts of whole genome data of cancer patients in comparison to control group such as 1000 Genomes.

We are cataloguing the altered palindromes in whole cancer genomes, and analysing the palindrome changes in transcription factor binding sites (TFBS) in both normal and breast tumors, and analyse whether they affect gene expression and function [30]. As TFBS motifs typically contain a palindromic sequence, alteration to these motifs or formation of motifs in new locations may alter or create a binding affinity for transcription factors and other proteins through mutations. This would provide a direction to understand how, through alterations to palindromes, genetic variants may contribute to chromosomal rearrangements and gene regulation defects that may eventually lead to breast cancer pathogenesis.
